# Risk factors for early-onset lung cancer in Korea: analysis of a nationally representative population-based cohort

**DOI:** 10.4178/epih.e2023101

**Published:** 2023-11-21

**Authors:** Jihun Kang, Taeyun Kim, Kyung-Do Han, Jin-Hyung Jung, Su-Min Jeong, Yo Hwan Yeo, Kyuwon Jung, Hyun Lee, Jong Ho Cho, Dong Wook Shin

**Affiliations:** 1 Department of Family Medicine, Kosin University Gospel Hospital, Kosin University College of Medicine, Busan, Korea; 2 Division of Pulmonology, Department of Internal Medicine, The Armed Forces Goyang Hospital, Goyang, Korea; 3 Department of Statistics and Actuarial Science, Soongsil University, Seoul, Korea; 4 Department of Medical Statistics, College of Medicine, The Catholic University of Korea, Seoul, Korea; 5 Department of Medicine, Seoul National University College of Medicine, Seoul, Korea; 6 Department of Family Medicine, Hallym University Sacred Heart Hospital, Dongtan, Korea; 7 Korea Central Cancer Registry, Division of Cancer Registration and Surveillance, National Cancer Center, Goyang, Korea; 8 Department of Internal Medicine, Hanyang University College of Medicine, Seoul, Korea; 9 Department of Thoracic and Cardiovascular Surgery, Samsung Medical Center, Sungkyunkwan University School of Medicine, Seoul, Korea; 10 Supportive Care Center, Samsung Comprehensive Cancer Center/Department of Family Medicine, Samsung Medical Center, Sungkyunkwan University School of Medicine, Seoul, Korea; 11 Department of Digital Health, SAIHST, Sungkyunkwan University, Seoul, Korea; 12 Center for Clinical Epidemiology, SAIHST, Sungkyunkwan University, Seoul, Korea

**Keywords:** Lung cancer, Early-onset lung cancer, Risk factors, Health behavior, Korea

## Abstract

**OBJECTIVES:**

We examined the associations of socioeconomic factors, health behaviors, and comorbidities with early-onset lung cancer.

**METHODS:**

The study included 6,794,287 individuals aged 20–39 years who participated in a Korean national health check-up program from 2009 to 2012. During the follow-up period, 4,684 participants developed lung cancer. Multivariable Cox regression analysis was used to estimate the independent associations of potential risk factors with incident lung cancer.

**RESULTS:**

Older age (multivariable hazard ratio [mHR], 1.13; 95% confidence interval [CI], 1.12 to 1.14) and female sex (mHR, 1.62; 95% CI, 1.49 to 1.75) were associated with increased lung cancer risk. Current smoking was also associated with elevated risk (<10 pack-years: mHR, 1.12; 95% CI, 1.01 to 1.24; ≥10 pack-years: mHR, 1.30; 95% CI, 1.18 to 1.45), but past smoking was not. Although mild alcohol consumption (<10 g/day) was associated with lower lung cancer risk (mHR, 0.92; 95% CI, 0.86 to 0.99), heavier alcohol consumption (≥10 g/day) was not. Higher income (highest vs. lowest quartile: mHR, 0.86; 95% CI, 0.78 to 0.94), physical activity for at least 1,500 metabolic equivalent of task-min/wk (vs. non-exercisers: mHR, 0.83; 95% CI, 0.69 to 0.99) and obesity (vs. normal weight: mHR, 0.89; 95% CI, 0.83 to 0.96) were associated with lower lung cancer risk, whereas metabolic syndrome was associated with increased risk (mHR, 1.13; 95% CI, 1.03 to 1.24).

**CONCLUSIONS:**

In young adults, age, female sex, smoking, and metabolic syndrome were risk factors for early-onset lung cancer, while high income, physical activity, and obesity displayed protective effects.

## INTRODUCTION

Lung cancer is the leading cause of cancer-specific death, and it ranks second in terms of cancer incidence in the United States and Korea [1,2]. Although tobacco smoking is commonly associated with the development of lung cancer, other risk factors such as advanced age [3–8], environmental and occupational exposure [9], and air pollution [10] contribute significantly to the incidence of this disease. However, given that lung cancer incidence sharply rises after the age of 50 years, most studies have focused on these risk factors in middle-aged to older patients. Consequently, a dearth of information is available on early-onset lung cancer, a topic that has garnered comparatively little research interest.

Assessing risk factors for early-onset lung cancer presents a challenge, given that incidence among young adults (those under 40 years of age) accounts for a mere 1.2% of total cases [7]. Several prior studies have consistently found that lung cancer in young adults is more common in female [6,7,11–14], primarily manifests as adenocarcinoma [6,7,11–13,15–17], and tends to present at an advanced stage [6,7,13,15] compared to lung cancer in older patients. However, these studies have primarily focused on comparing the clinical characteristics of young adults to those of older individuals [6,7,11–13,15–17], leaving a gap in the quantitative evaluation of risk factors associated with the development of lung cancer in the younger demographic.

A prior case-control study, matched for age and residence, proposed that heavy smoking, prolonged exposure to smoking, and a family history of lung cancer in first-degree relatives are risk factors for lung cancer in individuals under 45 years old [16]. While that study examined the relationships of tobacco smoking and other socioeconomic risk factors (such as education and marital status) with lung cancer among young adults, the low incidence of lung cancer cases in this age group impeded the ability to estimate other potential risk factors within a cohort design. These factors include alcohol consumption, physical activity, and cardiometabolic comorbidities. Furthermore, aside from studies of German (n=531) [16] and American samples using Surveillance, Epidemiology, and End Results (SEER) data (n=460,992) [6,7], most research on lung cancer in young adults has been based on data from single institutions. Consequently, nationwide data regarding risk factors associated with early lung cancer remain scarce.

In this context, we evaluated the risk factors of early-onset lung cancer, including socioeconomic elements, health behaviors, and comorbidities, using nationally representative population-based cohort data.

## MATERIALS AND METHODS

### Data sources and study participants

For the present study, we utilized a dataset from the Korean National Health Insurance Service (KNHIS). The KNHIS is a universal healthcare system that covers 97% of the Korean population. The KNHIS dataset contains all reimbursement information, which includes diagnoses based on the International Classification of Diseases, 10th revision (ICD-10) as well as diagnostic and treatment procedures, prescriptions, and medical costs incurred. Furthermore, data are included on the age, sex, insurance premium (as determined by income status), and any disabilities of the beneficiaries.

The KNHIS administers a biannual nationwide health check-up program for all Korean employees aged 20 years and older. This program includes anthropometric measurements, health behavior questionnaires (covering topics such as smoking status, alcohol consumption, and physical activity), and past medical history, as well as biochemical tests. These components collectively form the KNHIS screening database [18,19].

**Figure 1:**
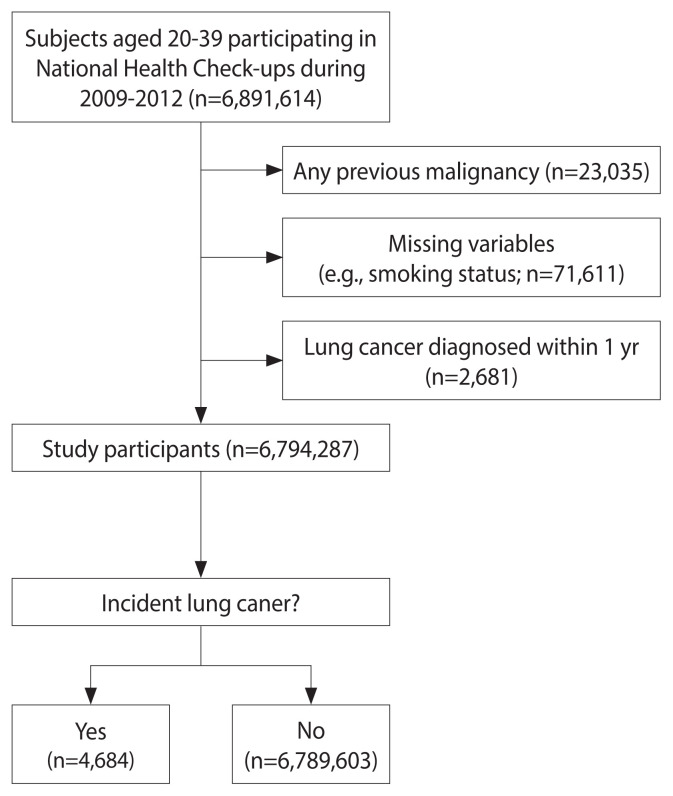
Flow diagram of study participants.

Between 2009 and 2012, a total of 6,891,614 individuals aged 20–39 years took part in nationwide health examinations. Of these, 97,326 participants were excluded for the following reasons: any prior diagnosis of malignancy (n=23,035), missing data on covariates (n=71,611), and a diagnosis of lung cancer or death within 1 year of enrollment (n=2,681). Consequently, the analysis included a total of 6,794,287 individuals (Figure 1).

### Outcome definition and follow-up

The primary outcome was defined as the incidence of lung cancer. Identification of lung cancer development was achieved using the lung cancer diagnosis code from the ICD-10 (C34), which was then matched to the critical illness registration program. This program requires a physician to submit a certificate of diagnosis for registration. Program beneficiaries can receive reductions in copayments of up to 95% for cancer diagnosis and treatment. As a result, these data offer a relatively high level of accuracy in cancer diagnosis [20] and have been utilized in various cancer epidemiology studies in Korea [8,21,22]. The study participants were monitored from the date of the first health examination (the index date) to the date of lung cancer diagnosis, the date of death from any other cause, or December 31, 2020, whichever occurred first.

### Socioeconomic and health behavior factors

Trained medical assistants collected anthropometric measurements, while data about health behaviors were gathered through a self-administered questionnaire [19]. Healthcare professionals measured height (in meters) and weight (in kilograms) during the screening examination. Body mass index (BMI) was calculated in kg/m^2^ and further categorized into 4 groups (<18.5, 18.5–22.9, 23.0–24.9, and ≥25.0 kg/m^2^). Participants with a BMI of ≥25.0 kg/m^2^ were considered to have obesity [23]. Income was divided into 4 groups based on insurance fees, which were determined by household income. Smoking status included categories of non-smoker, former smoker with <10 pack-years, former smoker with ≥10 pack-years, current smoker with <10 pack-years, and current smoker with ≥10 pack-years [24]. Alcohol consumption was also categorized, with options of non-drinker, <10.0 g/day, 10.0–19.9 g/day, 20.0–29.9 g/day, and ≥30.0 g/day [21]. The International Physical Activity Questionnaire was employed to assess physical activity levels. Exercise of light, moderate, and vigorous intensity was assigned 2.9, 4.0, and 7.0 metabolic equivalents of task (METs), respectively, to calculate total energy expenditure levels. Physical activity was then divided to establish 5 groups according to METs: non-exerciser, <500, 500–999, 1,000–1,499, and ≥1,500 MET-min/wk [25].

### Comorbidity factors

We collected information on comorbidities, such as disease codes and prescription records, over 1-year periods prior to the index date. Diabetes mellitus was identified by a diagnosis code record (E11.x–E14.x) in conjunction with antidiabetic medications or a fasting plasma glucose level of at least 126 mg/dL. Hypertension was defined through a combination of ICD-10 codes (I10–I13 and I15) and prescription records for antihypertensive agents, or a blood pressure reading of 140/90 mmHg or higher. Dyslipidemia was identified through a combination of ICD-10 codes (E78) and prescription records for lipid-lowering medications, or a total cholesterol level of 240 mg/dL or higher. Participants with metabolic syndrome were identified based on the criteria of the National Cholesterol Education Program’s Adult Treatment Panel III [26] and the abdominal obesity criteria of the Korean Society for the Study of Obesity [27]. A diagnosis of metabolic syndrome was made when 3 or more of the following were met: (1) a waist circumference of 90 cm or more in male and 85 cm or more in female; (2) a triglyceride level of 150 mg/dL or higher; (3) a high-density lipoprotein cholesterol level of less than 40 mg/dL in male and less than 50 mg/dL in female; (4) a blood pressure reading of 130/85 mmHg or higher, or current use of antihypertensive medications; and (5) a fasting glucose level of 100 mg/dL or higher, or current use of anti-diabetes medications.

### Statistical analysis

We utilized chi-square tests to compare the baseline characteristics of male and female for categorical variables and Student t-tests for continuous variables. The incidence rate of lung cancer was calculated as the total number of incident cases divided by 1,000,000 person-years. Multivariable Cox regression analysis was employed to estimate the independent associations of each potential risk factor with the onset of lung cancer. Given that metabolic syndrome is a composite of cardiovascular risk factors, we analyzed the associations of individual cardiovascular determinants and metabolic syndrome with the development of lung cancer in the following manner: model 1 incorporated age, sex, income, smoking status, alcohol consumption, physical activity, obesity, diabetes, hypertension, and dyslipidemia, while model 2 included age, sex, income, smoking status, alcohol consumption, physical activity, and metabolic syndrome. We presented the values derived from model 1 as the primary results of our analyses. To estimate potential interactive effects of age and sex with other covariates, stratification analyses were performed.

### Ethics statement

This study was approved by the Samsung Medical Center Institutional Review Board (2018-04-050). Due to the use of anonymized and de-identified data, the requirement for informed consent was waived.

## RESULTS

### Baseline characteristics of study participants

The median follow-up period for all participants was 9.62 years (interquartile range, 8.55–10.24), and the longest follow-up period was 11 years. The mean ages of participants with and without lung cancer were 33.6±4.5 years and 30.8±5.0 years, respectively (p<0.001). Those who developed lung cancer had a higher percentage of female compared to the control group (44.3 vs. 40.0%). Participants diagnosed with lung cancer exhibited higher rates of diabetes, hypertension, dyslipidemia, and metabolic syndrome than those in the control group. Furthermore, those who developed lung cancer were less physically active and consumed less alcohol compared to control participants (Table 1).

### Risk factors of lung cancer in young adults

#### Socioeconomic factors

Table 2 presents the factors associated with the risk of early-onset lung cancer. The risk of lung cancer was found to escalate by a factor of 1.13 for each year of increase in participant age (multivariable hazard ratio [mHR], 1.13; 95% confidence interval [CI], 1.12 to 1.14). Female displayed a higher risk of lung cancer than male (mHR, 1.58; 95% CI, 1.46 to 1.72). The risk of lung cancer was found to be lower in the higher income groups compared to the lowest income group (Q1: mHR, 0.86; 95% CI, 0.78 to 0.94; Q2: mHR, 0.85; 95% CI, 0.78 to 0.93).

#### Health behavior factors

Lung cancer risk was observed to be positively associated with current smoking relative to non-smoking status. Current smokers with ≥10 pack-years exhibited an mHR of 1.30 (95% CI, 1.18 to 1.45), while current smokers with <10 pack-years had an mHR of 1.12 (95% CI, 1.01 to 1.24). In contrast, past smoking was not associated with increased risk. Participants who consumed less than 10 g/day of alcohol displayed a lower risk of developing lung cancer than non-drinkers (mHR, 0.92; 95% CI, 0.86 to 0.99); however, this protective effect was not observed in participants who consumed 10 g/day or more of alcohol. Physically active participants were less likely to be diagnosed with lung cancer than non-exercisers, with an mHR of 0.88 for 1,000–1,499 MET-min/wk (95% CI, 0.78 to 0.99) and an mHR of 0.83 for 1,500 MET-min/wk or more (95% CI, 0.69 to 0.99).

#### Comorbidity factors

Individuals with obesity (BMI ≥25.0 kg/m^2^) demonstrated a lower risk of lung cancer relative to the group with a normal BMI (18.5–22.9 kg/m^2^), with an HR of 0.89 (95% CI, 0.83 to 0.96). No increased or decreased lung cancer risk was observed in the underweight (BMI <18.5 kg/m^2^) or overweight groups (BMI 23.0–24.9 kg/m^2^). Diabetes mellitus (mHR, 1.08; 95% CI, 0.90 to 1.30), hypertension (mHR, 1.10; 95% CI, 0.99 to 1.22), and dyslipidemia (mHR, 1.05; 95% CI, 0.94 to 1.17) showed no association with lung cancer risk. However, in model 2, metabolic syndrome was associated with an elevated risk of lung cancer (mHR, 1.13; 95% CI, 1.03 to 1.24).

### Risk stratification of lung cancer in young adults by age and sex

In age-stratified analyses, an interaction was observed between sex and age (p_interaction_=0.047). For female in their 20s, female sex demonstrated a stronger association with lung cancer risk (mHR, 1.83; 95% CI, 1.53 to 2.17) than for female in their 30s (mHR, 1.49; 95% CI, 1.36 to 1.64; p_interaction_=0.047). Significant interactive effects were noted for alcohol consumption (p_interaction_=0.011) and BMI (p_interaction_<0.001) with lung cancer risk in a sex-stratified analysis. Specifically, a protective association with mild drinking (<20 g/day) was found only in male (<10 g/day: mHR, 0.82; 95% CI, 0.74 to 0.91; ≥10 g/day: mHR, 0.87; 95% CI, 0.77 to 0.98), while being underweight was associated with elevated lung cancer risk in male only (mHR, 1.45; 95% CI, 1.16 to 1.81). Being overweight was associated with increased lung cancer risk only in female (mHR, 1.14; 95% CI, 1.01 to 1.30; Table 3).

## DISCUSSION

This research represents the largest cohort study to date to evaluate the socioeconomic factors, health behaviors, and comorbidities associated with lung cancer risk in young adults under 40 years old. The risk of lung cancer was found to increase with age, and female were observed to have a higher risk of lung cancer than male, particularly among younger participants. Current smoking was linked to an elevated risk of lung cancer, whereas past smoking was not. Mild alcohol consumption, defined as less than 10 g/day, appeared to have a protective effect against lung cancer, but heavier alcohol consumption, defined as 10 g/day or more, did not. Higher income, physical activity, and obesity were all associated with a reduced risk of lung cancer. Conversely, metabolic syndrome was linked to an elevated risk of developing lung cancer.

### Socioeconomic factors

In line with previous studies [3–8], advanced age was identified as a key determinant of lung cancer in young adults, with a 13% increase in risk for each additional year of age. The Liverpool Lung Project, a prospective cohort consisting of individuals aged 45 years and older, demonstrated that the risk of lung cancer rose by 4% for each 1-year increment in age (hazard ratio, 1.04; 95% CI, 1.02 to 1.06) [5]. Furthermore, the likelihood of lung cancer increased by 2% and 3% per year in American and Canadian cohorts, respectively (mean ages, 65.0 and 62.2 years, respectively) [28,29]. Consequently, while the absolute risk of lung cancer was lower in younger individuals compared to older people, the increase in relative risk per year was more pronounced in the younger population.

Among young adults, female exhibited a higher risk of lung cancer than male. This sex difference was especially pronounced in individuals under 30 years old. Many previous studies have noted that a higher proportion of female is a characteristic feature of early-onset lung cancer [6,7,11–13]. However, these studies have typically compared the risk of lung cancer in young individuals to that in older individuals, rather than comparing within an age group. A recent study utilizing SEER data from 2010 to 2014 reported that female under 50 years old displayed a higher incidence rate of lung cancer than male in the same age group (incidence rate ratio [IRR] for female vs. male, 1.13; 95% CI, 1.08 to 1.15) [14]. This difference was even more pronounced in cases of adenocarcinoma (IRRs for female vs. male, 1.29, 1.51). Given that adenocarcinoma is the predominant subtype at this age [6,7, 11–13,15–17] and is less influenced by smoking, the distinctive histopathological patterns of lung cancer could suggest an elevated risk of lung cancer in young female.

A negative social gradient was found to be associated with the risk of lung cancer. A previous meta-analysis revealed that the risk of lung cancer was 1.37 times higher (95% CI, 1.06 to 1.77) in the lowest compared to the highest income group [30]. Because income depends on occupational status, which is closely tied to health behaviors [31,32], low-income participants are more likely to smoke tobacco, consume unhealthy diets, be exposed to occupational carcinogens, and have limited access to health care. However, even after adjusting for smoking status, a negative social gradient in relation to lung cancer risk persisted, suggesting that disparities in socioeconomic status continue to independently contribute to lung cancer development. Moreover, sex-stratified analyses revealed stronger associations in male than in female participants, although these were only marginally significant. This suggests that occupational exposure (for example, factory work) may play a role in the link between income status and lung cancer risk. However, our data regarding job classification and occupational exposure are limited, and further studies are required.

### Health behavior factors

Smoking is a well-established risk factor for lung cancer [33]. In the present study, we found that tobacco exposure significantly contributed to the development of lung cancer in the young participants. Specifically, current smokers exhibited a risk elevated by 10–30%, depending on the pack-years of smoking. However, the effect size of smoking on lung cancer incidence was relatively small in young adults compared to older individuals. A Korean study involving participants aged 40 years and above found that those who smoked 10–20 pack-years had a 1.51-fold (95% CI, 1.22 to 1.88) higher risk of lung cancer compared to non-smokers, with the risk escalating with increased pack-years [8]. In terms of the contribution of smoking to lung cancer risk, the strength of this association was less pronounced in our study, which focused on a younger population (HR, 1.30; 95% CI, 1.18 to 1.45). The duration and amount of smoking are linked to lung cancer risk in a dose-dependent manner [16,33], and young adults inherently tend to have a shorter exposure period to smoking than older adults. This results in a smaller contribution of smoking to lung cancer risk.

In young adults who were former smokers, no significant increase was observed in the risk of lung cancer. This aligns with the findings of a previous study of British doctors, which suggested that ceasing to smoke in one’s 30s could eliminate over 90% of the risk associated with smoking [34]. Although a decade of follow-up is insufficient to estimate the lifelong risk reduction of early smoking cessation on lung cancer, our findings nonetheless underscore the importance of promoting early tobacco cessation to prevent an increased risk of lung cancer.

Our findings also suggest that moderate drinkers, specifically those who consume less than 10 g of alcohol per day, have a marginally reduced risk of lung cancer compared to non-drinkers. Individuals in good health may tend to consume alcohol in moderation, while those with medical issues often decrease their alcohol intake or cease consumption altogether. This correlation between health status and patterns of alcohol consumption could potentially account for the lower risk of lung cancer observed among mild alcohol drinkers [35].

Participants who were physically active demonstrated a reduced risk of lung cancer compared to those who were physically inactive. This finding aligns with a study conducted in the United States, which reported a reverse correlation between physical activity and lung cancer risk in participants aged 54–62 years [36]. Similarly, studies from the Female’s Health Initiative and Harvard University, involving participants aged 50–79 years and 39–88 years, respectively, found that physical activity was linked to a lower incidence of lung cancer in a dose-dependent manner [37,38]. Our study suggests that the protective correlation between physical activity and lung cancer incidence is not limited to older individuals, but also applies to younger people. From a biological perspective, it is reasonable to propose that physical activity may have a protective effect against lung cancer, as physical activity and exercise are known to enhance immune function [39]. Furthermore, increased pulmonary ventilation and perfusion could reduce the interactions between potential carcinogenic agents and the airways, thereby decreasing the incidence of lung cancer [40].

### Comorbidity factors

Obesity was found to have an inverse association with lung cancer risk. The literature indicates an intriguing relationship between obesity and the risk of incident lung cancer. While most previous cohort studies have suggested that a higher BMI is associated with a decreased risk of lung cancer [41,42], waist circumference, which is a proxy of central obesity, has been positively correlated with lung cancer risk [41]. Several hypotheses have been proposed to explain this paradoxical effect of obesity on the development of lung cancer. For instance, BMI has been inversely correlated with the level of oxidative DNA damage and carcinogen-DNA adducts in smokers, suggesting that a leaner physique may increase susceptibility to the development of smoking-related cancers [43,44]. Furthermore, a genetic variant of the *FTO* gene (rs9939609), which is associated with greater body weight, has been associated with lower lung cancer risk [45].

Notably, our study found an increased risk of lung cancer associated with metabolic syndrome, a finding that aligns with a recent cohort study. That study reported a linear association between the number of metabolic components and the development of lung cancer [24]. The degree of association between metabolic syndrome and lung cancer risk in our study was comparable to the overall effect previously found [24]. While the underlying mechanism remains unclear, insulin resistance and chronic inflammation are potential factors contributing to the heightened lung cancer risk observed in metabolic syndrome. Insulin resistance often leads to elevated serum insulin, which can enhance the effects of insulin-like growth factor 1. This factor may function in lung cancer development by promoting cell proliferation and angiogenesis [46]. Furthermore, chronic inflammation, caused by proinflammatory cytokines derived from adipocytes, could contribute to lung cancer development. This inflammation could inhibit DNA repair and create a microenvironment of genomic instability, leading to tumorigenesis [47].

In the present study, cardiometabolic comorbidities, such as hypertension, diabetes mellitus, and dyslipidemia, were not significant factors associated with lung cancer risk. However, hypertension was associated with an increased risk of lung cancer among male and individuals in their 30s. This finding aligns with a Finnish study that identified high blood pressure as an independent risk factor for incident lung cancer in male smokers with hypertension [48]. We also found hypertension to be a significant risk factor in young male, although we observed no significant or marginal sex difference (p_interaction_=0.07). However, a meta-analysis of randomized control studies has reported that antihypertensive medications did not significantly increase cancer risk [49]. Given the limited data and research on this topic, the reason for this positive association being observed only in male individuals remains unclear.

This study does present several limitations. First, the lack of histopathologic information in the KNHIS health check-ups and insurance claim data prevented us from classifying the subtypes of lung cancer. As a result, we were unable to investigate the differential risk factors associated with various histologic types of lung cancer. According to the national cancer registry data from Korea, adenocarcinoma accounted for 80% of all lung cancer cases in younger individuals [20] (Supplementary Material 1). This proportion exceeded the rates reported in other countries with comparable age groups, which range from 40% to 60% [6,7,16,17]. Second, we did not consider several factors that significantly contribute to the development of lung cancer at early ages, such as a family history of lung cancer, air pollution, and genetic mutations or polymorphisms. Third, our study sample had an over-representation of male participants (60% compared to 51.6% in census data). This discrepancy occurred because the study enrolled participants from a health screening program that was exclusively available to employees during the study period. Despite these limitations, we identified risk factors associated with lung cancer risk in a nationally representative sample of young adults. These risk factors could potentially serve as useful variables in risk stratification strategies for young individuals at high risk of lung cancer. This includes those with a positive family history of lung cancer, those exposed to occupational carcinogens, and those living in areas with high indoor radon levels.

In conclusion, the present study indicates that in young adults, age, female sex, smoking, and metabolic syndrome are risk factors for lung cancer, while higher income, physical activity, and obesity displayed inverse associations with lung cancer risk.

## Figures and Tables

**Table 1.** Baseline characteristics of study participants

Characteristics	Early-onset lung cancer incidence		p-value^1^
	Yes (n=4,684)	No (n=6,789,603)	
Age, mean±SD (yr)	33.6±4.5	30.8±5.0	<0.001
20-29	964 (20.6)	2,891,243 (42.6)	<0.001
30-39	3,720 (79.4)	3,898,360 (57.4)	
Sex			<0.001
Male	2,611 (55.7)	4,075,033 (60.0)	
Female	2,073 (44.3)	2,714,570 (40.0)	
Income			<0.001
Q1 (highest)	1,031 (22.0)	1,688,522 (24.9)	
Q2	1,188 (25.4)	2,096,156 (30.9)	
Q3	1,482 (31.6)	2,003,758 (29.5)	
Q4 (lowest)	983 (21.0)	1,001,167 (14.8)	
Smoking status (pack-yr)			<0.001
Non-smoker	2,585 (55.2)	3,716,798 (54.7)	
Former smoker with <10	492 (10.5)	700,679 (10.3)	
Former smoker with ≥10	183 (3.91)	349,020 (5.1)	
Current smoker with <10	742 (15.8)	1,210,833 (17.8)	
Current smoker with ≥10	682 (14.6)	812,273 (12.0)	
Alcohol consumption (g/day)			<0.001
Non-drinker	1,962 (41.9)	2,562,059 (37.7)	
<10.0	1,351 (28.8)	2,155,232 (31.7)	
10.0-19.9	636 (13.6)	991,387 (14.6)	
20.0-29.9	327 (7.0)	487,563 (7.2)	
≥30.0	408 (8.7)	593,362 (8.7)	
Physical activity (MET-min/wk)			<0.001
Non-exerciser	1,049 (22.4)	1,382,689 (20.4)	
<500	1,820 (38.9)	2,529,402 (37.3)	
500-999	1,324 (28.3)	2,032,503 (28.0)	
1,000-1,499	367 (7.8)	614,699 (9.1)	
≥1,500	124 (2.7)	230,310 (3.4)	
BMI (kg/m^2^)			0.222
<18.5	329 (7.0)	505,714 (7.5)	
18.5-22.9	2,181 (46.6)	3,164,194 (46.6)	
23.0-24.9	958 (20.5)	1,317,337 (19.4)	
≥25.0	1,216 (26.0)	1,802,358 (26.6)	
Diabetes	121 (2.6)	128,949 (1.9)	<0.001
Hypertension	426 (9.1)	502,257 (7.4)	<0.001
Dyslipidemia	391 (8.4)	459,750 (6.8)	<0.001
Metabolic syndrome	604 (12.9)	717,332 (10.6)	<0.001

Values are presented as number (%). SD, standard deviation; Q, quartile; MET, metabolic equivalents of task; BMI, body mass index.

1 Categorical variables were compared using the chi-square test, and continuous variables were compared using the Student t-test.

**Table 2.** Risk of lung cancer in young adults

Variables	n	Event (n)	Duration (PY)	Incidence rate (per 100,000 PY)	Univariable	Model 1^1^	Model 2^2^
Age							
Per 1 yr	-	-	-	-	1.13 (1.12, 1.13)	1.13 (1.12, 1.14)	1.13 (1.12, 1.14)
Sex							
Male	4,077,644	2,611	38,427,542.6	6.78	1.00 (reference)	1.00 (reference)	1.00 (reference)
Female	2,716,643	2,073	25,134,725.4	8.25	1.23 (1.16, 1.31)	1.58 (1.46, 1.72)	1.62 (1.49, 1.75)
Income							
Q1 (highest)	1,689,553	1,031	15,536,470.2	6.64	0.66 (0.61, 0.72)	0.86 (0.78, 0.94)	0.86 (0.78, 0.94)
Q2	2,097,344	1,188	19,459,692.7	6.11	0.61 (0.56, 0.66)	0.85 (0.78, 0.93)	0.85 (0.78, 0.93)
Q3	2,005,240	1,482	18,998,286.8	7.80	0.76 (0.70, 0.83)	0.92 (0.85, 1.00)	0.92 (0.85, 1.00)
Q4 (lowest)	1,002,150	983	9,567,818.3	10.27	1.00 (reference)	1.00 (reference)	1.00 (reference)
Smoking status (pack-yr)							
Non-smoker	3,719,383	2,585	34,596,750.0	7.47	1.00 (reference)	1.00 (reference)	1.00 (reference)
Former smoker with <10	701,171	492	6,657,102.4	7.39	0.97 (0.88, 1.07)	1.01 (0.91, 1.13)	1.01 (0.91, 1.13)
Former smoker with ≥10	349,203	183	3,243,215.8	5.64	0.76 (0.65, 0.88)	0.95 (0.81, 1.11)	0.95 (0.81, 1.11)
Current smoker with <10	1,211,575	742	11,376,708.6	6.52	0.87 (0.80, 0.94)	1.12 (1.01, 1.24)	1.12 (1.01, 1.24)
Current smoker with ≥10	812,955	682	7,688,491.1	8.87	1.17 (1.08, 1.27)	1.30 (1.18, 1.45)	1.29 (1.16, 1.44)
Alcohol consumption (g/day)							
Non-drinker	2,564,021	1,962	23,927,308.5	8.20	1.00 (reference)	1.00 (reference)	1.00 (reference)
<10.0	2,156,583	1,351	20,129,448.0	6.71	0.82 (0.76, 0.88)	0.92 (0.86, 0.99)	0.93 (0.86, 0.99)
10.0-19.9	992,023	636	9,339,152.5	6.81	0.83 (0.75, 0.90)	0.95 (0.87, 1.05)	0.95 (0.87, 1.05)
20.0-29.9	487,890	327	4,592,433.7	7.12	0.86 (0.77, 0.97)	0.96 (0.85, 1.09)	0.96 (0.85, 1.09)
≥30.0	593,770	408	5,573,925.3	7.32	0.89 (0.80, 0.99)	1.01 (0.90, 1.13)	1.00 (0.89, 1.13)
Physical activity (MET-min/wk)							
Non-exerciser	1,383,738	1,049	12,881,555.6	8.14	1.00 (reference)	1.00 (reference)	1.00 (reference)
<500	2,531,222	1,820	23,740,196.2	7.67	0.94 (0.87, 1.00)	0.97 (0.90, 1.04)	0.97 (0.90, 1.04)
500-999	2,033,827	1,324	19,014,025.4	6.96	0.85 (0.79, 0.92)	0.95 (0.88, 1.04)	0.95 (0.88, 1.03)
1,000-1,499	615,066	367	5,767,558.8	6.36	0.78 (0.69, 0.88)	0.88 (0.78, 0.99)	0.88 (0.78, 0.99)
≥1,500	230,434	124	2,158,931.9	5.74	0.70 (0.58, 0.85)	0.83 (0.69, 0.99)	0.82 (0.68, 0.99)
BMI (kg/m^2^)							
<18.5	506,043	329	4,696,833.9	7.01	0.95 (0.85, 1.07)	1.07 (0.95, 1.20)	1.07 (0.95, 1.20)
18.5-22.9	3,166,375	2,181	29,568,682.4	7.38	1.00 (reference)	1.00 (reference)	1.00 (reference)
23.0-24.9	1,318,295	958	12,394,350.4	7.73	1.04 (0.97, 1.13)	1.00 (0.92, 1.08)	1.00 (0.92, 1.08)
≥25.0	1,803,574	1,216	16,902,391.0	7.19	0.97 (0.91, 1.04)	0.89 (0.83, 0.96)	0.88 (0.81, 0.95)
Diabetes							
No	6,291,604	4,563	62,354,799.9	7.32	1.00 (reference)	1.00 (reference)	-
Yes	502,683	121	1,207,468.1	10.02	1.37 (1.14, 1.64)	1.08 (0.90, 1.30)	-
Hypertension							
No	6,334,146	4,258	58,837,673.6	7.24	1.00 (reference)	1.00 (reference)	-
Yes	460,141	426	4,724,594.4	9.02	1.24 (1.12, 1.37)	1.10 (0.99, 1.22)	-
Dyslipidemia							
No	6,076,351	4,293	59,230,914.6	7.25	1.00 (reference)	1.00 (reference)	-
Yes	717,936	391	4,331,353.4	9.03	1.24 (1.12, 1.37)	1.05 (0.94, 1.17)	-
Metabolic syndrome							
No	4,077,644	4,080	56,810,731.3	7.18	1.00 (reference)	-	1.00 (reference)
Yes	2,716,643	604	6,751,536.7	8.95	1.24 (1.14, 1.35)	-	1.13 (1.03, 1.24)

Values are presented as hazard ratio (95% confidence interval). PY, person-years; Q, quartile; MET, metabolic equivalents of task; BMI, body mass index.

1 Model 1 was adjusted for age, sex, income, smoking status, alcohol consumption, physical activity, obesity, diabetes, hypertension, and dyslipidemia.

2 Model 2 was adjusted for age, sex, income, smoking status, alcohol consumption, physical activity, and metabolic syndrome.

**Table 3.** Stratification of lung cancer risk by age and sex in young adults^1^

Variables	N	Age (yr)					Sex				
		Event (n)	20-29	Event (n)	30-39	p_interaction_	Event (n)	Male	Event (n)	Female	p_interaction_
Age											
Per 1 yr								1.13 (1.12, 1.14)		1.12 (1.11, 1.13)	0.204
Sex											
Male	4,077,644	398	1.00 (reference)	2,213	1.00 (reference)	0.047		-		-	
Female	2,716,643	566	1.83 (1.53, 2.17)	1,507	1.49 (1.36, 1.64)			-		-	
Income						0.817					0.070
Q1 (highest)	1,689,553	260	0.84 (0.66, 1.08)	771	0.87 (0.79, 0.96)		365	0.76 (0.67, 0.87)	666	1.00 (0.87, 1.16)	
Q2	2,097,344	359	0.82 (0.65, 1.05)	829	0.86 (0.78, 0.95)		601	0.81 (0.73, 0.91)	587	0.94 (0.82, 1.09)	
Q3	2,005,240	265	0.95 (0.73, 1.21)	1,217	0.91 (0.83, 0.99)		931	0.88 (0.80, 0.98)	551	1.01 (0.87, 1.17)	
Q4 (lowest)	1,002,150	80	1.00 (reference)	903	1.00 (reference)		714	1.00 (reference)	269	1.00 (reference)	
Smoking status (pack-yr)						0.124					0.530
Non-smoker	3,719,383	633	1.00 (reference)	1,952	1.00 (reference)		682	1.00 (reference)	1,903	1.00 (reference)	
Former smoker with <10	701,171	77	1.36 (1.06, 1.75)	415	0.95 (0.85, 1.08)		424	1.00 (0.88, 1.13)	68	1.07 (0.84, 1.37)	
Former smoker with ≥10	349,203	43	0.99 (0.72, 1.37)	140	0.93 (0.78, 1.11)		137	0.97 (0.81, 1.17)	46	0.89 (0.66, 1.20)	
Current smoker with <10	1,211,575	143	1.19 (0.95, 1.48)	599	1.10 (0.98, 1.23)		691	1.11 (1.00, 1.24)	51	1.33 (0.99, 1.76)	
Current smoker with ≥10	812,955	68	1.23 (0.92, 1.64)	614	1.29 (1.15, 1.44)		677	1.30 (1.16, 1.45)	5	0.80 (0.33, 1.92)	
Alcohol consumption (g/day)						0.088					0.011
Non-drinker	2,564,021	382	1.00 (reference)	1,580	1.00 (reference)		735	1.00 (reference)	1,227	1.00 (reference)	
<10.0	2,156,583	333	0.99 (0.85, 1.15)	1,018	0.90 (0.83, 0.98)		706	0.82 (0.74, 0.91)	645	1.02 (0.92, 1.12)	
10.0-19.9	992,023	113	0.86 (0.69, 1.07)	523	0.97 (0.87, 1.08)		500	0.87 (0.77, 0.98)	136	1.08 (0.90, 1.30)	
20.0-29.9	487,890	71	1.20 (0.92, 1.56)	256	0.91 (0.79, 1.05)		294	0.92 (0.80, 1.06)	33	0.79 (0.56, 1.12)	
≥30.0	593,770	65	0.89 (0.67, 1.18)	343	1.03 (0.90, 1.17)		376	0.96 (0.84, 1.09)	32	0.90 (0.62, 1.28)	
Physical activity (MET-min/wk)						0.763					0.147
Non-exerciser	1,383,738	183	1.00 (reference)	866	1.00 (reference)		506	1.00 (reference)	543	1.00 (reference)	
<500	2,531,222	369	1.01 (0.84, 1.20)	1,451	0.96 (0.88, 1.04)		965	1.03 (0.92, 1.14)	855	0.91 (0.82, 1.02)	
500-999	2,033,827	298	0.98 (0.81, 1.18)	1,026	0.95 (0.87, 1.04)		804	1.03 (0.92, 1.15)	520	0.88 (0.78, 0.99)	
1,000-1,499	615,066	89	1.03 (0.80, 1.33)	278	0.85 (0.74, 0.97)		252	0.98 (0.84, 1.14)	115	0.76 (0.62, 0.93)	
≥1,500	230,434	25	0.81 (0.54, 1.24)	99	0.83 (0.68, 1.03)		84	0.83 (0.66, 1.05)	40	0.88 (0.64, 1.22)	
BMI (kg/m^2^)						0.312					<0.001
<18.5	506,043	112	0.93 (0.76, 1.14)	217	1.13 (0.98, 1.31)		84	1.45 (1.16, 1.81)	245	0.97 (0.84, 1.11)	
18.5-22.9	3,166,375	538	1.00 (reference)	1,643	1.00 (reference)		907	1.00 (reference)	1,274	1.00 (reference)	
23.0-24.9	1,318,295	161	1.06 (0.88, 1.27)	797	0.99 (0.90, 1.08)		644	0.93 (0.84, 1.03)	314	1.14 (1.01, 1.30)	
≥25.0	1,803,574	153	0.87 (0.72, 1.05)	1,063	0.90 (0.83, 0.98)		976	0.88 (0.80, 0.97)	240	0.89 (0.77, 1.02)	
Diabetes						0.235					0.067
No	6,291,604	959	1.00 (reference)	3,604	1.00 (reference)		2,507	1.00 (reference)	2,056	1.00 (reference)	
Yes	502,683	5	0.64 (0.27, 1.54)	116	1.10 (0.91, 1.33)		104	1.17 (0.96, 1.43)	17	0.72 (0.45, 1.17)	
Hypertension						0.196					0.064
No	6,334,146	932	1.00 (reference)	3,326	1.00 (reference)		2,242	1.00 (reference)	2,016	1.00 (reference)	
Yes	460,141	32	0.88 (0.61, 1.26)	394	1.12 (1.01, 1.25)		369	1.15 (1.02, 1.29)	57	0.88 (0.67, 1.17)	
Dyslipidemia						0.923					0.787
No	6,076,351	928	1.00 (reference)	3,365	1.00 (reference)		2,309	1.00 (reference)	1,984	1.00 (reference)	
Yes	717,936	36	1.04 (0.74, 1.45)	355	1.05 (0.94, 1.17)		302	1.06 (0.94, 1.20)	89	1.03 (0.83, 1.27)	

Values are presented as hazard ratio (95% confidence interval). Q, quartile; MET, metabolic equivalents of task; BMI, body mass index.

1 The stratified analysis was adjusted for age, sex, income, smoking status, alcohol consumption, physical activity, obesity, diabetes, hypertension, and dyslipidemia.

## Data Availability

This study was performed using the National Health Insurance System database. However, the findings do not necessarily reflect the views of the National Health Insurance Corporation. Restrictions apply to the availability of these data, which were used under license for this study.
