# Supplementary material for: Risk factors for early-onset lung cancer in Korea: analysis of a nationally representative population-based cohort
Source: Epidemiol Health. 2023 Nov 21;45:e2023101. doi: 10.4178/epih.e2023101 (PMC10876445; doi:10.4178/epih.e2023101)
Supplement: Supplementary file 1 [file epih-45-e2023101-Supplementary-1.docx]

Supplementary Material 1. Adenocarcinoma in early lung cancer based on national cancer registry data of Korea (2015-2016)

|  | Total | 20-29 | 30-39 | p-value |
| --- | --- | --- | --- | --- |
| Adenocarcinoma, N(%) | 46 (80.7) | 8 (66.7) | 38 (84.4) | <0.001 |
| Non-adenocarcinoma, N (%) | 11 (19.3) | 4 (33.3) | 7 (15.6) |  |

p-value was calculated using Chi-square test
